# Supplementary material for: Sexual dysfunctions related to use of antipsychotics: A protocol for a systematic review and meta-analysis
Source: PLoS One. 2025 Aug 21;20(8):e0329559. doi: 10.1371/journal.pone.0329559 (PMC12370141; doi:10.1371/journal.pone.0329559)
Supplement: S1 Appendix — (PDF) [file pone.0329559.s002.pdf]

Appendix S1

to *'Sexual difficulty related to use of antipsychotics: a protocol for systematic review and meta-analysis'*

PubMed

("Antipsychotic agents" OR "Antipsychotic agents" [tiab:~3] OR "Antipsychotic agents" [majr] OR neuroleptic* OR antipsychotic* OR "anti-psychotic"[tiab] OR "Dopamine Antagonists"[Mesh] OR "Dopamine Atagonists" [tiab:~3] OR "Dopamine Antagonists" OR "Chlorpromazine"[MesH] OR chlorpromazine* OR "Droperidol"[MesH] OR droperidol* OR "Fluphenazine"[MesH] OR fluphenazine* OR "Haloperidol"[MesH] OR haloperidol* OR "Loxapine"[MesH] OR loxapine* OR "Molindone"[MesH] OR molindone* OR "Perphenazine"[MesH] OR perphenazine* OR "Pimozide"[MesH] OR pimozide* OR "Prochlorperazine"[MesH] OR prochlorperazine* OR "Thiothixene"[MesH] OR thiothixene* OR tiotixene* OR "Thioridazine"[MesH] OR thioridazine* OR "Trifluoperazine"[MesH] OR trifluoperazine* OR "Aripiprazole"[MesH] OR aripiprazole* OR Asenapine[nm] OR asenapine* OR Brexpiprazole[nm] OR brexpiprazole* OR Cariprazine[nm] OR cariprazine* OR "Clozapine"[MesH] OR clozapine* OR Iloperidone[nm] OR iloperidone* OR "Lurasidone hydrochloride"[MesH] OR lurasidone* OR Olanzapine[nm] OR olanzapine* OR "Paliperidone palmitate"[MesH] OR paliperidone* OR "Quetiapine fumarate"[MesH] OR "Quetiapine fumarate" OR quetiapine* OR "Risperidone"[MesH] OR risperidone* OR Ziprasidone[nm] OR ziprasidone*)

AND

("Sexual Dysfunctions, Psychological"[majr] OR "Sexual Dysfunctions, Psychological"[tiab] OR "Sexual dysfunction"[tiab:~3] OR sexual function*[tiab] OR "hypoactive sexual desire disorder"[tiab] OR "HSDD"[tiab] OR "low libido"[tiab] OR "high libido"[tiab] OR "low sexual drive"[tiab] OR "high sexual drive"[tiab] OR "female sexual interest/arousal disorder"[tiab] OR "Sexual Arousal Disorder"[tiab] OR "Female Orgasmic Disorder"[tiab] OR "Male Orgasmic Disorder"[tiab] OR Dysfunction[tiab] OR vaginismus[tiab] OR "genito-pelvic pain/penetration disorder"[tiab] OR GPPPD[tiab] OR "orgasmic dysfunction"[tiab] OR "delayed orgasm*"[tiab] OR "sexual desire"[tiab] OR "premature ejaculation*"[tiab] OR "ejaculation praecox"[tiab] OR "erectile disorder*"[tiab] OR "Erectile dysfunction*"[tiab] OR hypersexual*[tiab] OR "sexual complaint*"[tiab] OR "sexual side effects"[tiab])

Embase

(Antipsychotic NEAR/3 agent OR ‘anti-psychotic’**:ab,ti** OR ‘neuroleptic agent'/mj OR 'dopamine receptor blocking agent'/mj OR 'chlorpromazine'/exp OR 'droperidol'/exp OR 'fluphenazine'/exp OR 'haloperidol'/exp OR 'loxapine'/exp OR 'molindone'/exp OR 'perphenazine'/exp OR 'pimozide'/exp OR 'prochlorperazine'/exp OR 'tiotixene'/exp OR 'thioridazine'/exp OR thiothixene OR 'trifluoperazine'/exp OR 'aripiprazole'/exp OR 'asenapine'/exp OR 'brexpiprazole'/exp OR 'cariprazine'/exp OR 'clozapine'/exp OR 'iloperidone'/exp OR 'lurasidone'/exp OR 'Lurasidone hydrochloride' OR 'olanzapine'/exp OR 'paliperidone'/exp OR 'quetiapine'/exp OR 'risperidone'/exp OR 'ziprasidone'/exp)

AND

('psychosexual disorder'/exp OR psychosexual NEAR/3 disorder OR 'psychosexual disorder'/mj OR 'sexual dysfunction'/mj OR 'sexual function*'**:ab,ti** OR 'hypoactive sexual desire disorder'/exp OR libido/exp OR hsdd/exp OR 'libido disorder'/exp OR 'sexual arousal disorder'/exp OR 'orgasm disorder'/exp OR 'delayed orgasm'/exp OR 'anorgasmia'/exp OR 'sexual desire'/exp OR 'dyspareunia'/exp OR 'female sexual dysfunction'/exp OR frigidity/exp OR 'male sexual dysfunction'/exp OR vaginism/exp OR vaginismus/exp OR 'genito-pelvic pain/penetration disorder'/exp OR gpppd/exp OR 'sexual dysfunction'/exp OR 'premature ejaculation'/exp OR 'erectile dysfunction'/exp OR hypersexuality/exp OR 'ejaculation praecox'**:ab,ti**)

PsyInfo

(MM "Neuroleptic Drugs" OR "Antipsychotic agents" OR OR antipsychotic* OR Anti-psychotic* OR neuroleptic OR "Dopamine Antagonists" OR DE "Chlorpromazine" OR droperidol OR DE "Fluphenazine" OR DE "Haloperidol" OR DE "Loxapine" OR DE "Molindone" OR DE "Perphenazine" OR DE "Pimozide" OR DE "Prochlorperazine" OR DE "Thiothixene" OR tiotixene OR DE "Thioridazine" OR asenapine OR brexpiprazole OR cariprazine OR DE "Clozapine" OR iloperidone OR OR lurasidone OR DE "Olanzapine" OR "Paliperidone palmitate" OR paliperidone OR "Quetiapine fumarate" OR DE "Quetiapine" OR DE "Risperidone" OR Ziprasidone)

AND

(MM "Sexual Function Disturbances" OR MM OR Sexual N3 Function* OR "Inhibited Sexual Desire" OR HSDD OR MM "Female Sexual Dysfunction" OR (("Libido" OR "sexual drive") AND (high and low)) OR "female sexual interest/arousal disorder" OR "Sexual Arousal Disorder" OR "Female Orgasmic Disorder" OR "Male Orgasmic Disorder" OR "orgasmic dysfunction" OR "delayed orgasm*" OR "sexual desire" OR DE "Dyspareunia" OR DE "Vaginismus" OR "genito-pelvic pain/penetration disorder" OR GPPPD OR DE "Premature Ejaculation" OR MM "Erectile Dysfunction" OR "erectile disorder*" OR hypersexual)

WOS

TS="Antipsychotic agents"
TS="neuroleptic agent"

ALL=("hypoactive sexual desire disorder" OR "HSDD" OR (("Libido" OR "sexual drive") AND (high and low)) OR "female sexual interest/arousal disorder" OR "Sexual Arousal Disorder" OR "Female Orgasmic Disorder" OR "Male Orgasmic Disorder" OR "orgasmic dysfunction" OR "delayed orgasm*" OR “sexual function*” OR "sexual desire" OR dyspareunia OR vaginismus OR "genito-pelvic pain/penetration disorder" OR "GPPPD" OR "Sexual Dysfunction, Physiological" OR premature ejaculation* OR "ejaculation praecox" OR "erectile disorder*" OR "Erectile dysfunction*" OR hypersexual*)

#3 OR #2 OR #1

TS="Sexual Dysfunctions, Psychological"

TS="orgasmic dysfunction"

TS="Orgasmic Disorder"

ALL=(antipsychotic* OR neuroleptic* OR Dopamine Antagonists OR Chlorpromazine OR droperidol* OR fluphenazine* OR haloperidol* OR loxapine* OR molindone* OR perphenazine* OR pimozide* OR prochlorperazine* OR thiothixene* OR tiotixene* OR thioridazine* OR trifluoperazine* OR aripiprazole* OR asenapine* OR brexpiprazole* OR cariprazine* OR clozapine* OR iloperidone* OR lurasidone* OR olanzapine* OR "Paliperidone palmitate" OR paliperidone* OR "Quetiapine fumarate" OR quetiapine* OR "Risperidone" OR risperidone* OR Ziprasidone OR ziprasidone*)

#5 OR #6 OR #7 OR #8

#4 AND #9

CINHAL

(MM "Antipsychotic Agents+" OR Neuroleptic N3 Drug OR antipsychotic* OR neuroleptic OR Anti-psychotic* OR MH "Dopamine Antagonists+" OR MH "Chlorpromazine" OR MH "Droperidol" OR MH "Fluphenazine" OR MH "Haloperidol" OR MH "Loxapine" OR "molindone" OR "perphenazine" OR "pimozide" OR MH "Prochlorperazine" OR MH "Thiothixene" OR "tiotixene" OR MH "Thioridazine" OR "trifluoperazine" OR MH "Aripiprazole" OR MH "Asenapine" OR "brexpiprazole" OR "cariprazine" OR MH "Clozapine" OR MH "Iloperidone" OR "lurasidone" OR MH "Olanzapine+" "Paliperidone palmitate" OR MH "Paliperidone" OR MH "Quetiapine" OR "Quetiapine fumarate" OR MH "Quetiapine" OR MH "Risperidone" OR MH "Ziprasidone")

AND

(MH "Sexual Desire Disorders+" OR MH "Hypoactive Sexual Desire Disorder" OR "HSDD" OR ((MH "Libido" OR "sexual drive") AND (high and low)) OR Sexual N3 function* OR MH "Sexual Dysfunction, Female+" OR MH "Sexual Dysfunction, Male+" OR "Sexual Arousal Disorder" OR MH "Sexual Desire Disorders+" OR "Orgasmic Disorder" OR "orgasmic dysfunction" "delayed orgasm" OR "anorgasmia" OR "sexual desire" OR MH "Dyspareunia" OR "genito-pelvic pain/penetration disorder" OR "GPPPD" OR MH "Premature Ejaculation" OR "ejaculation praecox" OR "erectile disorder*" OR MH "Erectile Dysfunction" OR hypersexual*)

Search Name:

Date Run: 30/07/2024 21:37:44

Comment:

ID Search Hits

#1 MeSH descriptor: [Dopamine Antagonists] explode all trees 615

#2 MeSH descriptor: [Antipsychotic Agents] explode all trees 6270

#3 ((antipsychotic* OR anti-psychotic* OR neuroleptic* OR Dopamine Antagonists OR Chlorpromazine OR droperidol* OR fluphenazine* OR haloperidol* OR loxapine* OR molindone* OR perphenazine* OR pimozide* OR prochlorperazine* OR thiothixene* OR tiotixene* OR thioridazine* OR trifluoperazine* OR aripiprazole* OR asenapine* OR brexpiprazole* OR cariprazine* OR clozapine* OR iloperidone* OR lurasidone* OR olanzapine* OR "Paliperidone palmitate" OR paliperidone* OR "Quetiapine fumarate" OR quetiapine* OR "Risperidone" OR risperidone* OR Ziprasidone OR ziprasidone*)) 23749

#4 #1 OR #2 OR #323749

#5 MeSH descriptor: [Sexual Dysfunction, Physiological] explode all trees 3034

#6 MeSH descriptor: [Sexual Dysfunctions, Psychological] explode all trees 2847

#7 MeSH descriptor: [Vaginismus] explode all trees 16

#8 MeSH descriptor: [Dyspareunia] explode all trees 311

#9 MeSH descriptor: [Premature Ejaculation] explode all trees 155

#10 MeSH descriptor: [Erectile Dysfunction] explode all trees 1951

#11 (("hypoactive sexual desire disorder" OR HSDD OR Sexual NEXT function* OR (("Libido" OR "sexual drive") AND (high and low)) OR "female sexual interest/arousal disorder" OR "Sexual Arousal Disorder" OR Sexual NEAR/3 Dysfunction OR "Female Orgasmic Disorder" OR "Male Orgasmic Disorder" OR "orgasmic dysfunction" OR "delayed orgasm" OR anorgasmia OR "genito-pelvic pain/penetration disorder" OR "GPPPD" OR "ejaculation praecox" OR "erectile disorder")) 7853

#12 #5 OR #6 OR #7 OR #8 OR #9 OR #10 OR #11 9788

#13 #4 AND #12 303

Medline EBSCO

(MM "Antipsychotic Agents" OR antipsychotic* OR anti-psychotic* OR neuroleptic OR MM "Dopamine Antagonists+" OR MH "Chlorpromazine" OR MH "Droperidol" OR MH "Fluphenazine" OR MH "Haloperidol" OR MH "Loxapine" OR "molindone" OR "perphenazine" OR "pimozide" OR MH "Prochlorperazine" OR MH "Thiothixene" OR "tiotixene" OR MH "Thioridazine" OR "trifluoperazine" OR MH "Aripiprazole" OR MH "Asenapine" OR "brexpiprazole" OR "cariprazine" OR MH "Clozapine" OR MH "Iloperidone" OR "lurasidone" OR MH "Olanzapine+" "Paliperidone palmitate" OR MH "Paliperidone" OR MH "Quetiapine" OR "Quetiapine fumarate" OR MH "Quetiapine" OR MH "Risperidone" OR MH "Ziprasidone")

AND

(MH "Sexual Dysfunctions, Psychological+" OR "Hypoactive Sexual Desire Disorder" OR HSDD OR ((MH Libido OR "sexual drive") AND (high and low)) OR "Sexual Dysfunction, Female" OR "Sexual Dysfunction, male" OR "Sexual Desire Disorders" OR Sexual N3 function* OR "Sexual Arousal Disorder" OR ""Orgasmic Disorder" OR "orgasmic dysfunction" "delayed orgasm" OR "anorgasmia" OR "sexual desire" OR MH "Dyspareunia" OR genito-pelvic pain/penetration disorder" OR "GPPPD" OR MH "Premature Ejaculation" OR "ejaculation praecox" OR "erectile disorder*" OR MH "Erectile Dysfunction+")

EUROPEPMC

("Antipsychotic agents" OR antipsychotic* OR ant-psychotic* OR neuroleptic* OR "Dopamine Antagonists" OR Chlorpromazine OR droperidol* OR fluphenazine* OR haloperidol* OR loxapine* OR molindone* OR perphenazine* OR pimozide* OR prochlorperazine* OR thiothixene* OR tiotixene* OR thioridazine* OR trifluoperazine* OR aripiprazole* OR asenapine* OR brexpiprazole* OR cariprazine* OR clozapine* OR iloperidone* OR lurasidone* OR olanzapine* OR "Paliperidone palmitate" OR paliperidone* OR "Quetiapine fumarate" OR quetiapine* OR Risperidone OR risperidone* OR Ziprasidone)

AND

("Sexual Dysfunctions, Psychological" OR "hypoactive sexual desire disorder" OR "HSDD" OR (("Libido" OR "sexual drive") AND (high and low)) OR "female sexual interest/arousal disorder" OR "Sexual Arousal Disorder" OR "Female Orgasmic Disorder" OR "Male Orgasmic Disorder" OR "orgasmic dysfunction" OR "delayed orgasm*" OR "sexual desire" OR dyspareunia OR vaginismus OR "genito-pelvic pain/penetration disorder" OR "GPPPD" OR "Sexual function*" OR "sexual dysfunction*" OR "premature ejaculation*" OR "ejaculation praecox" OR "erectile disorder*" OR "Erectile dysfunction*" OR hypersexual*)

Medline OVID

Query Results from 24 Jul 2024

1 exp *Antipsychotic Agents/ or exp *Dopamine Antagonists/ or exp Chlorpromazine/ or exp Droperidol/ or exp Fluphenazine/ or exp Haloperidol/ or exp Loxapine/ or exp Molindone/ or exp Perphenazine/ or exp Pimozide/ or exp Prochlorperazine/ or exp Thiothixene/ or exp Thioridazine/ or exp Trifluoperazine/ or exp Aripiprazole/ or exp Clozapine/ or exp Lurasidone Hydrochloride/ or exp Olanzapine/ or exp Paliperidone Palmitate/ or exp Quetiapine Fumarate/ or exp Risperidone/ 108,087

2 (asenapine or cariprazine or iloperidone or Ziprasidone OR anti-psychotic* OR antipsychotic*) 160

3 1 or 2 108,109

4 exp *Sexual Dysfunctions, Psychological/ or exp Erectile Dysfunction/ or exp *Sexual Dysfunctions, Psychological/ or exp Dyspareunia/ or exp Vaginismus/ 30,646

5 ("hypoactive sexual desire disorder" or HSDD or "Sexual Dysfunction*" or "low libido" or "high libido" or "low sexual drive" or "high sexual drive" or "female sexual interest/arousal disorder" or "Sexual Arousal Disorder" or "genito-pelvic pain/penetration disorder" or GPPPD or ejaculation praecox or "erectile disorder*" or hypersexual*).mp. [mp=title, book title, abstract, original title, name of substance word, subject heading word, floating sub-heading word, keyword heading word, organism supplementary concept word, protocol supplementary concept word, rare disease supplementary concept word, unique identifier, synonyms, population supplementary concept word, anatomy supplementary concept word]24,703

6 4 or 5 46,848

7 3 and 6 485
