# Supplementary material for: Sexual dysfunctions related to use of antipsychotics: A protocol for a systematic review and meta-analysis
Source: PLoS One. 2025 Aug 21;20(8):e0329559. doi: 10.1371/journal.pone.0329559 (PMC12370141; doi:10.1371/journal.pone.0329559)
Supplement: Attachment — Submitted filename: Recommendations_AK.docx [file pone.0329559.s003.docx]

**Title: Sexual difficulty related to use of antipsychotics: a protocol for systematic review and meta-analysis**

**Introduction:**

- Clarify how the current review differs from the 2011 meta-analysis in terms of scope, the inclusion of newer antipsychotic drugs, and updated research findings.
- Include a clear, operational definition of "sexual dysfunction," as it is central to the focus of the review.
- Report available prevalence statistics of sexual dysfunction among patients taking antipsychotics to highlight its clinical importance.
- Provide brief explanations of terms such as *hyperprolactinemia* and *dopamine antagonists* to ensure accessibility for interdisciplinary readers, especially those from behavioral sciences and therapy-based backgrounds.

**Methodology:**

- Define “primary mental health disorder” with an operational definition and clarify which psychiatric conditions are included. Although diagnoses are mentioned, it is not clearly specified what is meant by “primary mental health disorder” in the context of the study.
- Specify whether the study accounts for the duration of antipsychotic use in inclusion or exclusion criteria.
- Indicate if the study considers or stratifies participants based on gender, given its relevance to sexual dysfunction.
- Clearly define the inclusion and exclusion criteria in terms of age, gender, and specific diagnoses.
- Justify the inclusion of serum prolactin as an outcome variable and explain its role as a physiological correlate of sexual dysfunction.

**Exclusion Criteria:**

- Clarify whether neurological conditions, substance use disorders, hypogonadism, and androgen disorders are explicitly excluded, and if they are not already captured under "somatic diseases."

**Search Strategy:**

- Mention if MeSH terms will be used in the systematic search strategy to ensure thorough and standardized literature retrieval.

**Data Extraction and Analysis:**

- Recommend including variables such as sample size per study, treatment or follow-up duration, and sex/gender distribution in the descriptive statistics to enhance the comprehensiveness of the analysis.

**Discussion:**

- Strengthen the rationale by clearly linking how the findings could influence clinical decisions, improve adherence to antipsychotic treatment, and guide future intervention strategies.
