# Supplementary material for: Sexual dysfunctions related to use of antipsychotics: A protocol for a systematic review and meta-analysis
Source: PLoS One. 2025 Aug 21;20(8):e0329559. doi: 10.1371/journal.pone.0329559 (PMC12370141; doi:10.1371/journal.pone.0329559)
Supplement: Attachment — Submitted filename: Response to Reviewers.docx [file pone.0329559.s005.docx]

Dear Editor,

We thank you and the reviewers for the thorough and constructive feedback on our manuscript titled ‘*Sexual difficulty related to use of antipsychotics: a protocol for a systematic review and meta-analysis’*.

We would like to express our sincere gratitude to the reviewers and the editorial team for their insightful and constructive feedback. The comments provided were instrumental in refining the scope, enhancing the methodological rigor, and improving the clarity and overall quality of our manuscript. We have carefully considered each suggestion and made corresponding revisions throughout the protocol. These include clarifications of key definitions, refinements to the inclusion criteria, additional detail in our analytical plan, and a more thorough discussion of the clinical implications of our findings. We have also explicitly addressed potential sources of bias and expanded upon our strategy for ensuring methodological consistency and transparency.

We appreciate the opportunity to revise our work and have addressed all comments stepwise in detail below. Reviewer comments are reproduced in bold, followed by our responses. *The corresponding text of the manuscript is in italic, all revisions made in the manuscript and this rebuttal letter are highlighted in yellow for ease of reference.*

**Reviewer #1**

**1. Inclusion of Open-Label Trials**
**The inclusion of open-label trials raises concerns about potential bias, particularly given the subjective nature of sexual dysfunction assessments. Unblinded studies may lead to over- or underreporting of symptoms due to patient and clinician expectations. The authors should clarify their rationale for including open-label trials and discuss whether any sensitivity analyses are planned to assess their impact on the findings.**

**Response:**
Thank you for this important point. We agree that open-label trials carry an inherent risk of bias, particularly for subjective outcomes like sexual dysfunction. We chose to include open-label trials to ensure a comprehensive synthesis of the available evidence, given the limited number of double-blind studies in this area. Furthermore, we’ve already planned to conduct subgroup analyses to examine the influence of (patient) blinding on our primary and secondary outcomes.

‘*Only blinded and open randomized controlled trials will be included. Open-label randomized trials will be included in this meta-analysis to ensure comprehensive coverage of the available evidence, particularly given the limited number of double-blind randomized controlled trials assessing sexual dysfunction in patients treated with antipsychotics. In the case of a crossover study, only the first phase will be considered for data-extraction. All other study designs will be excluded.’* (now in the section Study Design, page 10, line 189-192)

*‘The sensitivity of our findings will be further evaluated by conducting analyses restricted to: (1) studies with complete outcome data (i.e., no imputation performed by the primary authors), (2) studies in which we did not impute response data based on means and SD’s, (3) studies with comparable antipsychotic dosages across arms, (4) studies providing unpublished data, (5) studies assessed as having a low risk of bias according to the predefined risk of bias criteria, (6) blinded trials, and (7) direct head-to-head trials only.’* (now in the section Pair-wise meta-analysis, page 17-18, line 384-389)

*‘To ensure the robustness of different medical treatments on our outcomes, we will conduct subgroup analyses based on the following characteristics: (1) baseline levels of sexual function, (2) blinding, (3) type of psychiatric disorder, (4) concomitant with antidepressants, (5) gender and (6) age.’* (now in the section Network meta-analysis, page 19 , line 422-425 )

**2. Confounding Effects of Psychiatric Diagnoses**
**“The protocol includes patients with bipolar disorder and major depressive disorder, both of which can independently influence sexual function. While the authors plan subgroup analyses based on concomitant antidepressant use, it is unclear whether stratification or adjustments for psychiatric disorders are planned. Clarifying how the study will account for these confounding effects would strengthen the methodology.”**

**Response:**
We agree that psychiatric diagnoses can confound experiencing of sexual dysfunction. Therefore, we have now clarified in the protocol that subgroup and meta-regression analyses will be conducted based on psychiatric diagnosis to explore their potential modifying effects on outcomes. Additionally, we will stratify analyses based on diagnosis (e.g., schizophrenia vs. bipolar disorder vs. major depressive disorder) when data availability permits.

*‘**To ensure the robustness of different medical treatments on our outcomes, we will conduct subgroup analyses based on the following characteristics: (1) baseline levels of sexual function, (2) blinding, (3) type of psychiatric disorder, (4) concomitant with antidepressants, (5) gender and (6) age.’* (now in the section Network meta-analysis, page 19, line 422-425)

**3. Consistency in Study Inclusion Criteria**
**In the Abstract, the authors state that both “controlled and uncontrolled randomized trials” will be included, while in the Methods, they specify “only blinded and open randomized controlled trials.” If the intention is to include only RCTs, the wording in the Abstract should be clarified to avoid potential confusion regarding whether non-controlled trials are eligible.**

**Response:**
Thank you for pointing this out. We have revised the abstract to clarify that only randomized controlled trials—both blinded and open-label—are eligible for inclusion. Non-randomized or uncontrolled trials will be excluded to ensure methodological consistency.

*‘Studies will be included if they meet predefined inclusion criteria, which include only controlled randomized trials assessing sexual functioning in patients receiving antipsychotic treatment.’* (text to be found in the Abstract, page 3, line 45)

*‘Only blinded and open randomized controlled trials will be included. Open-label randomized trials will be included in this meta-analysis to ensure comprehensive coverage of the available evidence, particularly given the limited number of double-blind randomized controlled trials assessing sexual dysfunction in patients treated with antipsychotics.’* (text to be found in the section Study design, page 10, line 189-192)

**Reviewer #2**

**You have obtained many results. Write a conclusion based on these results, in a broad manner.**

**Response:**
Thank you for your helpful comment. We have revised both the Discussion and Conclusion sections to address your suggestion and to better reflect the implications of our findings.

*‘By systematically comparing the sexual side effects and prolactin-related impacts of various antipsychotics, this study aims to provide clinicians with evidence-based guidance for selecting medications that minimize sexual dysfunction, a key factor affecting patient adherence. Improved understanding of these side effect profiles can enhance shared decision-making, support personalized treatment plans, and ultimately improve treatment adherence and quality of life(2). Furthermore, the findings may inform the development of targeted interventions and monitoring strategies to mitigate sexual dysfunction, thereby optimizing long-term psychiatric care.’* (text to be found in the Discussion, page 20-21, line 460-467)

*‘The findings of this meta-analysis can directly inform clinical practice guidelines by providing robust, comparative evidence on the sexual side effect profiles of various antipsychotic medications. Incorporating these results into treatment recommendations will enable clinicians to better balance efficacy and tolerability when selecting antipsychotics, particularly for patients at risk of or experiencing sexual dysfunction. This evidence-based approach supports personalized medicine by guiding prescribers toward options with lower sexual adverse effects, potentially enhancing patient adherence and satisfaction. Furthermore, the integration of these findings into clinical guidelines can promote shared decision-making between clinicians and patients, fostering informed discussions about treatment choices and potential side effects. Ultimately, this will contribute to improved long-term outcomes by reducing treatment discontinuation related to sexual dysfunction and enhancing overall quality of life.*’ (text to be found in the Conclusion, page 22, line 502-512 )

**Reviewer #3**

**1. Clarify how the current review differs from the 2011 meta-analysis in terms of scope, the inclusion of newer antipsychotic drugs, and updated research findings.**
Thank you very much for this important point. We have now explicitly clarified in the Introduction how our review expands upon the 2011 meta-analysis by including newer antipsychotic medications approved since then, incorporating updated research findings, and broadening the scope to include both subjective sexual dysfunction and biological markers.

*‘In 2011, a meta-analysis on sexual dysfunction in patients with mental health disorders using antipsychotics was performed and showed that different antipsychotics influence various dimensions of sexual function(25). Since then, significant advancements have occurred, including the approval and widespread use of newer antipsychotic agents, alongside a growing body of research investigating their side effect profiles. Given these developments, there is a clear need to update and expand upon the previous synthesis(27). This updated review differs from the 2011 meta-analysis by incorporating recent studies, including newer antipsychotics, and by broadening the scope to include biological correlates such as prolactin. Furthermore, our meta-analysis is based exclusively on controlled studies, enhancing methodological rigor and minimizing bias. By focusing on randomized controlled trials, this review aims to provide more robust and clinically relevant estimates of antipsychotic-induced sexual dysfunction.’* (text to be found in the Introduction, page 8, line 141-150)

**2. Include a clear, operational definition of "sexual dysfunction," as it is central to the focus of the review.**
We appreciate this insightful suggestion. To enhance clarity, we have provided a precise operational definition of “sexual dysfunction” early in the manuscript, which reflects its multidimensional clinical presentation as assessed by validated instruments.

*‘Sexual dysfunction is a clinically significant impairment in sexual desire, arousal, orgasm, or satisfaction, as measured by validated instruments and reported by the patient as causing distress or functional impairment in the context of sexual activity .(3).’* (text to be found in the Introduction, page 5, line 70-73)

**3. Report available prevalence statistics of sexual dysfunction among patients taking antipsychotics to highlight its clinical importance.**
Thank you for this helpful recommendation. We have added up-to-date prevalence data from recent epidemiological studies in the Introduction to underscore the clinical significance of sexual dysfunction associated with antipsychotic treatment

*‘The prevalence of sexual dysfunction among individuals using antipsychotic medication is estimated to range 16-60%, depending on the specific agent, assessment method, and population studied(8, 9).’* (text to be found in the Introduction, page 5, line 80-82)

**4. Provide brief explanations of terms such as hyperprolactinemia and dopamine antagonists to ensure accessibility for interdisciplinary readers.**
We appreciate the suggestion to improve accessibility for a broader audience. Consequently, concise explanations of key terms including hyperprolactinemia and dopamine antagonists have been incorporated.

*‘Although the group of antipsychotics is pharmacologically heterogeneous, they all have an affinity for the dopamine system, either as partial agonists or full dopamine antagonists,* *which are agents that block dopamine receptors—primarily D2 receptors—to inhibit dopaminergic neurotransmission. The intensity of dopamine blockade and whether an antipsychotic is only blocking dopamine (antagonists) or also have their own dopaminergic activities (partial agonists) cause differential effects on the dopamine system and possibly the associated influence on sexual (dys)function.’* (text to be found in the Introduction, page 6, line 97-101)

*‘Effects of antipsychotics on the dopamine receptors in the pituitary interfere with physiologic dopaminergic modulation, resulting in increased or decreased prolactin secretion in peripheral blood(5). Hyperprolactinemia (abnormally elevated level of serum prolactin) can lead to hypogonadism, reduced testosterone levels, and disruption of the hypothalamic-pituitary-gonadal axis.’* (text placed in the Introduction, page 6, line 110)

**5. Define “primary mental health disorder” with an operational definition and clarify which psychiatric conditions are included.**
Thank you for highlighting this need for precision. An operational definition of “primary mental health disorder” is now included, specifying the diagnostic categories covered and the clinical rationale for their inclusion in this study.

*‘Studies will be included if they involve patients diagnosed with a primary mental health disorder, defined as the foremost, clinically significant psychiatric condition currently affecting the individual’s functioning and constituting the main focus of diagnosis or treatment, requiring antipsychotic medication. This includes psychotic disorders (including schizophrenia), depressive disorders, bipolar disorder, obsessive-compulsive disorder, anxiety disorders, and post-traumatic stress disorder (PTSD), diagnosed according to DSM-III(-R), DSM-IV(-TR), DSM-5(-TR), ICD-10, or ICD-11 criteria.’* ( in the section Inclusion criteria, page 10, line 197-203)

**6. Specify whether the study accounts for the duration of antipsychotic use in inclusion or exclusion criteria.**
We thank the reviewer for this comment. We clarified the inclusion criteria in terms of duration of antipsychotic use.

*‘Both antipsychotic-naive patients and patients with a history of antipsychotic use will be included, in order to capture a representative clinical population and reflect real-world prescribing practices.* *All patients will initiate treatment with an antipsychotic that they were not using immediately prior to study entry, ensuring a clearly defined treatment start point for all participants.’* (text to be found in the section Inclusion criteria, page 10, line 203-207)

**7. Indicate if the study considers or stratifies participants based on gender, given its relevance to sexual dysfunction.**
Thank you for emphasizing this critical factor. Gender will be reported in the descriptive characteristics of included studies and will also be incorporated into subgroup analyses to examine sex-specific differences in effects of antipsychotics on sexual dysfunction outcomes.

*‘Participant demographics: Data on participant sex (% female), mean age, diagnosis, baseline prolactin levels, baseline sexual difficulties, and use of concomitant antidepressants.’* (in the section Data extraction process, page 14, line 298-300)

*‘To ensure the robustness of different medical treatments on our outcomes, we will conduct subgroup analyses based on the following characteristics: (1) baseline levels of sexual function, (2) blinding, (3) type of psychiatric disorder, (4) concomitant with antidepressants, (5) gender, and (6) age.’* (in the section Network meta-analysis, page 19, line 422-425)

**8. Clearly define the inclusion and exclusion criteria in terms of age, gender, and specific diagnoses.**
We appreciate this suggestion for improving clarity. The inclusion and exclusion criteria are now explicitly detailed regarding age, gender, and diagnostic categories.

*‘Studies will be included if they involve patients diagnosed with a primary mental health disorder, defined as the foremost, clinically significant psychiatric condition currently affecting the individual’s functioning and constituting the main focus of diagnosis or treatment, requiring antipsychotic medication. This includes psychotic disorders (including schizophrenia), depressive disorders, bipolar disorder, obsessive-compulsive disorder, anxiety disorders, and post-traumatic stress disorder (PTSD), diagnosed according to DSM-III(-R), DSM-IV(-TR), DSM-5(-TR), ICD-10, or ICD-11 criteria.* *Both antipsychotic-naive patients and patients with a history of antipsychotic use will be included, in order to capture a representative clinical population and reflect real-world prescribing practices.* *Furthermore, the included patients in these studies must be 18 years or older and both female and male patients are included.‘* (in the section Inclusion criteria, page 10, line 197-207)

**9. Justify the inclusion of serum prolactin as an outcome variable and explain its role as a physiological correlate of sexual dysfunction.**
Thank you for this valuable comment. We would like to note that the introduction already includes an explanation of why serum prolactin is a relevant biological marker in the context of antipsychotic-induced sexual dysfunction. Additionally, we have provided a definition of hyperprolactinemia to clarify its clinical relevance.

*‘Prolactin secretion is regulated by the modulation of pituitary dopamine 2 (D2) receptors, with dopamine inhibiting prolactin secretion.* *Antagonistic effects of antipsychotics on the dopamine receptors in the pituitary interferes with physiologic dopaminergic modulation, resulting in increased prolactin secretion in peripheral blood(5). Hyperprolactinemia (abnormally elevated level of serum prolactin) can lead to hypogonadism, reduced testosterone levels, and disruption of the hypothalamic-pituitary-gonadal axis. Prolactin elevation as well as its secondary disruption of the hypothalamic-pituitary-gonadal axis is thought to contribute to decreased sexual desire, sexual arousal, anorgasmia, amenorrhea, infertility, gynecomastia, and galactorrhoea(19). However, newer antipsychotics with partial agonistic effects on the dopamine system may hardly influence or even decrease prolactin secretion.’* (in the Introduction, page 6, line 110)

**10. Clarify whether neurological conditions, substance use disorders, hypogonadism, and androgen disorders are explicitly excluded, and if they are not already captured under "somatic diseases."**
We appreciate this helpful suggestion. The exclusion criteria have been refined to explicitly mention these conditions for improved clarity.

*‘Studies will exclude patients diagnosed with concomitant somatic diseases known to cause sexual dysfunction or difficulty (e.g. neurological conditions, diabetes mellitus, thyroid disease, renal disease, hypogonadism, androgen disorders, etc.), as well as patients using multiple antipsychotic agents. Studies involving postpartum patients will also be excluded, as this condition can significantly impact sexual function(28).* *Substance use is not considered an exclusion criterion, as excluding these patients would significantly limit the generalizability of findings and is often not feasible due to high comorbidity rates in clinical populations treated with antipsychotics(29)*.’ (in the section Exclusion criteria, page 10-11, line 210-217)

**11. Mention if MeSH terms will be used in the systematic search strategy to ensure thorough and standardized literature retrieval.**
Thank you for pointing this out. We confirm that MeSH terms will be incorporated into our search strategy.

*‘Outcome measures for sexual functioning will include (MeSH) terms such as sexual functioning or performance, sexual desire (disorders), sexual arousal (disorders), and orgasmic (disorders) along with related terms (see supplementary appendix S1)(2, 5).’* (in the section Inclusion criteria, page 11, line 227)

**12. Recommend including variables such as sample size per study, treatment or follow-up duration, and sex/gender distribution in the descriptive statistics.**
We appreciate this practical suggestion. We incorporated sample size data based on the reviewer’s suggestion, while trial duration (follow-up duration) and sex/gender distribution were already included in our descriptive analyses.

*‘Study characteristics: Information such as the first author, year of publication, journal, sample size, country where the study was conducted, study design (e.g., non-blinded), name of the questionnaire used to assess sexual functioning, trial duration (follow-up), and sponsorship.’ (now in the section Data extraction process, page 13, line 294-297)*

*‘Participant demographics: Data on participant sex (% female), mean age, diagnosis, baseline weight, baseline prolactin levels, baseline sexual difficulties, and use of concomitant antidepressants*.’ (now in the section Data extraction process, page 14, line 294-297)

**13. Strengthen the rationale by clearly linking how the findings could influence clinical decisions, improve adherence to antipsychotic treatment, and guide future intervention strategies.**
Thank you for highlighting the importance of clinical relevance. We have altered the Discussion and Conclusion to better articulate how our findings could inform clinical practice, improve patient adherence, and guide future research and intervention development.

*‘By systematically comparing the sexual side effects and prolactin-related impacts of various antipsychotics, this study aims to provide clinicians with evidence-based guidance for selecting medications that minimize sexual dysfunction, a key factor affecting patient adherence. Improved understanding of these side effect profiles can enhance shared decision-making, support personalized treatment plans, and ultimately improve treatment adherence and quality of life(2). Furthermore, the findings may inform the development of targeted interventions and monitoring strategies to mitigate sexual dysfunction, thereby optimizing long-term psychiatric care.’* (now in the Discussion, page 20-21, line 460-467)

*‘Furthermore, the integration of these findings into clinical guidelines can promote shared decision-making between clinicians and patients, fostering informed discussions about treatment choices and potential side effects. Ultimately, this will contribute to improved long-term outcomes by reducing treatment discontinuation related to sexual dysfunction and enhancing overall quality of life.’* (now in the Conclusion, page 22, line 507-512)

**Reviewer #4**

**1. Inclusion Criteria and Data Sources
The protocol excludes studies using semi-structured interviews or non-validated tools, focusing only on standardized questionnaires. While this increases internal validity, it risks excluding clinically meaningful data, especially qualitative insights. Recommendation: Consider adding a sensitivity analysis including studies with semi-structured interviews or discuss this limitation more explicitly. You might also explore incorporating grey literature systematically (e.g., OpenGrey, ProQuest Dissertations) to mitigate publication bias*.***

**Response:**
We thank the reviewer for highlighting this important point. We have acknowledged in the revised manuscript that excluding studies using semi-structured interviews or non-validated tools may limit the inclusion of qualitative insights and reduce comprehensiveness. Given the focus on quantitative synthesis and comparability of drugs and across studies, we have prioritized validated and standardized questionnaires to maximize validity and reduce heterogeneity. However, following your suggestion, we now explicitly discuss this limitation and its implications.

‘*Studies reporting sexual dysfunction rates based on spontaneous reports or semi-structured interviews lacking validated and standardized assessment tools will be excluded. This approach ensures the use of validated and standardized questionnaires, thereby enhancing the internal validity of the analysis and minimizing heterogeneity across included studies, which is essential for robust quantitative synthesis and comparability.’* (text to be found in the section Outcomes, page 11-12, line 237-242)

*‘The use of subjective self-reported questionnaires, although validated, may introduce recall bias. Consequently, some nuances related to patient-reported experiences and context-specific factors might not be fully captured, which could limit the comprehensiveness of the findings. Another limitation of this review is the exclusion of studies that rely on spontaneous reports or semi-structured interviews without the use of validated and standardized instruments for assessing the prevalence of sexual dysfunction. While this decision enhances the internal validity and comparability of included studies by reducing heterogeneity, it may also result in the omission of potentially valuable qualitative data and clinically relevant insights.’* (in the Discussion, page 21-22, line 484-491)

**2. Translation of Non-English Articles
The authors plan to use ChatGPT 4.0 for machine translation of articles. This raises concerns about translation accuracy and loss of nuance. Recommendation: Provide a clear quality control plan, e.g., using back-translation or secondary human verification, especially for key methodological sections.**

**Response:**
We appreciate this valuable concern regarding translation accuracy. To address this, we have implemented a quality control plan whereby key sections (such as methods and results) translated via ChatGPT will undergo secondary verification by a bilingual researcher fluent in the respective language.

*‘For eligible articles published in languages unfamiliar to the reviewers, machine translation tools (e.g., ChatGPT 4.0 or newer versions) will be employed, complemented by back-translation or secondary human verification of critical methodological sections to ensure accuracy and preserve the integrity of the data.’ (*in the section Search Strategy, page 9, line 182-185)

**3. Handling Missing Data
The protocol outlines the use of Furukawa’s method for imputing response rates, which is valid under certain assumptions. Recommendation: Clarify the assumptions under which this method will be applied and plan sensitivity analyses to test robustness with and without imputed data.**

**Response:**
Thank you for this recommendation. We have clarified that Furukawa’s imputation method will be applied when response rates are lacking and only means +- SDs are reported by assuming a normal distribution of the rating scale. We will perform sensitivity analyses on the impact of studies in the analyses in which data was imputed on the conclusions drawn.

*‘When the number of patients experiencing sexual difficulty is not directly reported but means (with SD or SE) for baseline and endpoint data on a sexual difficulty rating scale are available, Furukawa et al.’s method will be applied to estimate the number of affected patients(32). Furukawa et al found that assuming normal distributions, while often such symptom measures are skewed and not normally distributed, did not affect the conclusions drawn. We will perform sensitivity analyses to assess the impact of including studies in the analyses in which data was imputed.*’ (in the section Measurement of (adverse) effects on sexual function, page 15, line 320-326)

In the section ‘pair-wise meta-analysis’ we added this analyses. *‘The sensitivity of our findings will be further evaluated by conducting analyses restricted to: (1) studies with complete outcome data (i.e., no imputation performed by the primary authors), (2) studies in which we did not impute response data based on means and SD’s, (3) studies with comparable antipsychotic dosages across arms, (4) studies providing unpublished data, (5) studies assessed as having a low risk of bias according to the predefined risk of bias criteria, (6) blinded trials, and (7) direct head-to-head trials only.’* (in the section Pair-wise meta-analysis, page 17-18, line 384-389)

**4. Transitivity and Effect Modifiers in NMA
Although the authors mention assessing transitivity, the specific clinical and methodological effect modifiers are not clearly listed. Recommendation: Explicitly describe variables (e.g., baseline sexual function, diagnosis, age, gender, medications, dosage) that will be evaluated to ensure comparability across trials.**

**Response:**
We appreciate the reviewer’s valuable suggestion. To improve clarity, these variables have now been explicitly described and highlighted in yellow in the revised manuscript.

*‘We will focus on: sex, mean age, diagnosis, baseline prolactin levels, baseline sexual difficulties, use of concomitant antidepressants, dosage of anti-psychotic medication, type of dosage (flexible or fixed), and duration of use.’* (in Network Meta-analysis, page 17-18, line 384-389)

**5. Subgroup Analyses and Meta-Regression
Subgroup and metaregression analyses are briefly mentioned. Recommendation: Specify the exact variables to be included, criteria for inclusion (e.g., minimum number of studies), and how multicollinearity will be managed.**

**Response:**
We appreciate this constructive input. We have expanded our protocol to specify planned subgroup and meta-regression variables, including gender, diagnosis, publication year, dosage, treatment duration, and study quality. No strict minimum number of studies will be required for variable inclusion; instead, variables will be selected based on their clinical or methodological relevance and data availability. To address multicollinearity, variance inflation factors will be examined, and highly correlated variables will be excluded or combined accordingly. These details are now outlined in the revised manuscript.

‘*Additionally, we will explore whether treatment effects for the two primary outcomes are robust in subgroup analyses and network meta-regression using the following study and participant characteristics: (1) publication year, (2) psychiatric diagnosis (3), industry sponsorship, (4) treatment duration, and (5) gender distribution. The sensitivity of our findings will be further evaluated by conducting analyses restricted to: (1) studies with complete outcome data (i.e., no imputation performed by the primary authors), (2) studies in which we did not impute response data based on means and SD’s, (3) studies with comparable antipsychotic dosages across arms, (4) studies providing unpublished data, (5) studies assessed as having a low risk of bias according to the predefined risk of bias criteria, (6) blinded trials, and (7) direct head-to-head trials only.’ (in the section Pair-wise meta-analysis, page 17-18, line 380-389 )*

*‘To address potential multicollinearity among covariates in the network meta-regression analyses, we will examine variance inflation factors (VIFs). Variables exhibiting high collinearity (i.e., VIF values exceeding acceptable thresholds) will be either excluded from the model or combined with related variables, as appropriate, to preserve model stability and interpretability.*’ (now in the section Pair-wise meta-analysis, page 18, line 391-395)

**6. Potential Bias and Publication Bias
While funnel plots and selection models are planned, no mention is made of bias introduced by industry sponsorship or selective reporting. Recommendation: Include plans to evaluate sponsorship bias explicitly and consider including funding sources as a moderator in metaregression.**

**Response:**
We appreciate the reviewer’s insightful comment regarding the potential influence of industry sponsorship on study outcomes. In response, we clarify that funding source, including industry sponsorship, will be systematically evaluated as part of the risk of bias assessment. Specifically, studies with potential conflicts of interest or industry ties will be carefully appraised using established tools (e.g., the Cochrane Risk of Bias tool), and sponsorship will be considered when judging the overall quality of evidence. The industry sponsorship will be used for subgroup analyses and will also be used in the sensitivity analyses as these will be performed on examining the difference in outcomes if only studies with low risk of bias are included.

*‘We will address the potential biases in the following areas: the generating of the allocation sequence, concealing the allocation, blinding of both study personnel and participants, blinding of outcome reviewers, management of attrition, selective reporting of outcomes, imputation techniques used, and other relevant factors, including potential sponsorship bias.*’ (in the section Risk of Bias assessment, page 16, line 351)

*‘Additionally, we will explore whether treatment effects for the two primary outcomes are robust in subgroup analyses and network meta-regression using the following study and participant characteristics: (1) publication year, (2) psychiatric diagnosis (3), industry sponsorship, (4) treatment duration, and (5) gender distribution. The sensitivity of our findings will be further evaluated by conducting analyses restricted to: (1) studies with complete outcome data (i.e., no imputation performed by the primary authors), (2) studies in which we did not impute response data based on means and SD’s, (3) studies with comparable antipsychotic dosages across arms, (4) studies providing unpublished data, (5) studies assessed as having a low risk of bias according to the predefined risk of bias criteria, (6) blinded trials, and (7) direct head-to-head trials only.’* (in the section Pair-wise meta-analysis, page 17-18, line 380-389)

**7. Implementation in Clinical Practice
The discussion touches on shared decision-making but lacks details on how results will translate to practice. Recommendation: Expand the discussion on how the findings could inform treatment guidelines, shared decision-making tools, or clinical decision support systems.**

**Response:**
We appreciate this suggestion and have expanded the Discussion and Conclusion section to better articulate how our findings may inform clinical guidelines by identifying antipsychotics with more favourable sexual side effect profiles.

*‘By systematically comparing the sexual side effects and prolactin-related impacts of various antipsychotics, this study aims to provide clinicians with evidence-based guidance for selecting medications that minimize sexual dysfunction, a key factor affecting patient adherence. Improved understanding of these side effect profiles can enhance shared decision-making, support personalized treatment plans, and ultimately improve treatment adherence and quality of life(2). Furthermore, the findings may inform the development of targeted interventions and monitoring strategies to mitigate sexual dysfunction, thereby optimizing long-term psychiatric care.’* (in the Discussion, page 20-21, line 460-467)

*‘The findings of this meta-analysis can directly inform clinical practice guidelines by providing robust, comparative evidence on the sexual side effect profiles of various antipsychotic medications. Incorporating these results into treatment recommendations will enable clinicians to better balance efficacy and tolerability when selecting antipsychotics, particularly for patients at risk of or experiencing sexual dysfunction. This evidence-based approach supports personalized medicine by guiding prescribers toward options with lower sexual adverse effects, potentially enhancing patient adherence and satisfaction. Furthermore, the integration of these findings into clinical guidelines can promote shared decision-making between clinicians and patients, fostering informed discussions about treatment choices and potential side effects. Ultimately, this will contribute to improved long-term outcomes by reducing treatment discontinuation related to sexual dysfunction and enhancing overall quality of life.*’ (in the Conclusion, page 22, line 501-512)

**Minor Comments**

**1. Typographical Errors
Line 36: "prolactin levels land sexual dysfunction" should be "and sexual dysfunction." Multiple minor grammatical errors (e.g., "Antipsychotics are thought affect" should be "thought to affect"). Recommendation: A thorough language check is recommended to improve clarity*.***

**Response:**
Thank you for pointing out these errors. We have carefully proofread and corrected all typographical and grammatical mistakes throughout the manuscript to enhance clarity and readability.

**2. Figure and Supplementary Materials
Ensure that Figure 1 (decision-tree) and supplementary files (search strategy, PRISMA-P checklist) are well-integrated and referenced properly in the main text.**

**Response:**
We have reviewed the manuscript and ensured that Figure 1 and all supplementary materials are properly referenced and seamlessly integrated within the main text.

**3. References
The reference list is up-to-date and balanced; however, maybe these could enrich the discussion:**

- ***Clayton, A. H., & Montejo, A. L. (2014). Major depressive disorder, antidepressants, and sexual dysfunction. Journal of Clinical Psychiatry, 75(4), 383–391.*** [***https://doi.org/10.4088/JCP.13com13629***](https://doi.org/10.4088/JCP.13com13629)
- ***Ebrahim, S., Akl, E. A., Mustafa, R. A., Sun, X., Walter, S. D., & Heels-Ansdell, D. (2013). Addressing continuous data measured with different instruments for outcome synthesis in systematic reviews: A survey of current practice and methods. Systematic Reviews, 2, Article 70.*** [***https://doi.org/10.1186/2046-4053-2-70***](https://doi.org/10.1186/2046-4053-2-70)

**Response:**
*Thank you for these valuable suggestions. We have incorporated the first reference into the Discussion to further support our rationale.*

We are confident that the revised manuscript has been substantially strengthened thanks to your input, and we hope it now meets the expectations for publication. Once again, we thank you for your time, expertise, and commitment to supporting the development of high-quality research.

Sincerely,

Thalia Herder
On behalf of all authors
