# Supplementary material for: Sexual dysfunctions related to use of antipsychotics: A protocol for a systematic review and meta-analysis
Source: PLoS One. 2025 Aug 21;20(8):e0329559. doi: 10.1371/journal.pone.0329559 (PMC12370141; doi:10.1371/journal.pone.0329559)
Supplement: Attachment — Submitted filename: Response to Reviewers (second revision).docx [file pone.0329559.s006.docx]

Dear Editor,

We thank you for the opportunity to submit a second revision of our manuscript titled *“Sexual dysfunctions related to use of antipsychotics: a protocol for a systematic review and meta-analysis.”* We are grateful for the continued constructive feedback from the reviewer and the editorial team, which has helped us further improve the clarity and quality of our protocol.

We have addressed all comments carefully. Revisions include clarified terminology, refined definitions, and minor corrections to improve consistency and readability. Changes are highlighted in yellow in the manuscript and explained below.

**Reviewer #4**

We sincerely thank you for taking the time to re-evaluate our revised manuscript and for your encouraging and constructive comments. We deeply appreciate your acknowledgment of the substantial improvements, particularly regarding the clarification of inclusion/exclusion criteria, the enhanced statistical analysis plan, and our strategies for assessing bias and transitivity in the network meta-analysis (NMA). In response to your minor recommendations, we have made the following changes:

**Ensure consistent use of terms: e.g., there are instances of “sexual difficulties” vs. “sexual dysfunction”, using one term throughout would improve clarity**.
We have reviewed the manuscript thoroughly and replaced all instances of “sexual difficulties” with the term “sexual dysfunction” to ensure consistent terminology throughout the title and text. As this change affected multiple sections across the entire manuscript, highlighting each individual replacement in yellow would have resulted in excessive markup and reduced readability. Therefore, this global edit has not been highlighted in detail.

**In the introduction, the authors mention “sexual difficulties” affecting the “specific phases of sexual function”; consider clarifying early that this refers to desire, arousal, and orgasm to set context.**

In the Introduction section, we have clarified that the phrase “specific phases of sexual function” refers to *desire, arousal,* and *orgasm*. To provide immediate context for the reader, we included this clarification in the first paragraph of the Introduction, using the framework of the sexual response cycle. This ensures that the terminology is clear from the outset and aligns with established clinical and research definitions. We believe this clarification is sufficient and appropriately placed.

*‘Sexual dysfunction is a clinically significant impairment in sexual desire, arousal, orgasm, or satisfaction, as measured by validated instruments and reported by the patient as causing distress or functional impairment in the context of sexual activity(3).’* (in the Introduction, page 5, line 70-73)

**Double-check minor typographical issues like “land sexual dysfunction” in the abstract (should be “and sexual dysfunction”).**
We have corrected the typographical error in the abstract from “land sexual dysfunction” to “and sexual dysfunction. In addition, we have carefully reviewed the entire manuscript for any remaining typographical or formatting errors.

*‘Additionally, the analysis will explore the relationship between prolactin levels and sexual dysfunction.’* (now in the Abstract, page 3, line 37-28)

Once again, we truly appreciate your valuable feedback and the time you have invested in reviewing our protocol a second time. Your suggestions have helped improve the clarity and quality of our manuscript, and we hope the revised version meets your expectations.

Kind regards,

Thalia Herder
On behalf of all co-authors
